# Spleen regeneration after subcutaneous heterotopic autotransplantation in a mouse model

**DOI:** 10.1186/s40659-023-00427-4

**Published:** 2023-03-29

**Authors:** Andrey Elchaninov, Polina Vishnyakova, Anastasiya Lokhonina, Viktoria Kiseleva, Egor Menyailo, Maria Antonova, Aiaz Mamedov, Irina Arutyunyan, Galina Bolshakova, Dmitry Goldshtein, Xuhui Bao, Timur Fatkhudinov, Gennady Sukhikh

**Affiliations:** 1 Laboratory of Regenerative Medicine, National Medical Research Center for Obstetrics, Gynecology and Perinatology Named after Academician V.I. Kulakov of Ministry of Healthcare of Russian Federation, Moscow, Russia; 2 Laboratory of Growth and Development, Avtsyn Research Institute of Human Morphology of FSBI Petrovsky National Research Centre of Surgery, Moscow, Russia; 3 Histology Department, Medical Institute, Peoples’ Friendship University of Russia (RUDN University), Moscow, Russia; 4 Histology Department, Pirogov Russian National Research Medical University, Ministry of Healthcare of the Russian Federation, Moscow, Russia; 5 Laboratory of Stem Cells Genetics, Research Center of Medical Genetics, Moscow, Russia; 6 Institute of Therapeutic Cancer Vaccines, Fudan University Pudong Medical Center, Shanghai, China

**Keywords:** Spleen, Regeneration, Transplantation, Lymphocytes, Macrophages, Hematopoietic cells

## Abstract

**Background:**

Splenectomy may lead to severe postoperative complications, including sepsis and cancers. A possible solution to this problem is heterotopic autotransplantation of the spleen. Splenic autografts rapidly restore the regular splenic microanatomy in model animals. However, the functional competence of such regenerated autografts in terms of lympho- and hematopoietic capacity remains uncertain. Therefore, this study aimed to monitor the dynamics of B and T lymphocyte populations, the monocyte-macrophage system, and megakaryocytopoiesis in murine splenic autografts.

**Methods:**

The model of subcutaneous splenic engraftment was implemented in C57Bl male mice. Cell sources of functional recovery were studied using heterotopic transplantations from B10-GFP donors to C57Bl recipients. The cellular composition dynamics were studied by immunohistochemistry and flow cytometry. Expression of regulatory genes at mRNA and protein levels was assessed by real-time PCR and Western blot, respectively.

**Results:**

Characteristic splenic architecture is restored within 30 days post-transplantation, consistent with other studies. The monocyte-macrophage system, megakaryocytes, and B lymphocytes show the highest rates, whereas the functional recovery of T cells takes longer. Cross-strain splenic engraftments using B10-GFP donors indicate the recipient-derived cell sources of the recovery. Transplantations of scaffolds populated with splenic stromal cells or without them afforded no restoration of the characteristic splenic architecture.

**Conclusions:**

Allogeneic subcutaneous transplantation of splenic fragments in a mouse model leads to their structural recovery within 30 days, with full reconstitution of the monocyte-macrophage, megakaryocyte and B lymphocyte populations. The circulating hematopoietic cells provide the likely source for the cell composition recovery.

**Supplementary Information:**

The online version contains supplementary material available at 10.1186/s40659-023-00427-4.

## Background

A number of pathological conditions, such as traumatic injuries, thrombocytopenia and portal hypertension, may require splenectomy [1]. Despite its straightforwardness, this surgical procedure may entail longer-term complications that manifest 2–10 years post-surgery [1] as severe infectious processes [1] and increased cancer risks [2–4], indicating significant imbalances in both humoral and cellular immunity. The high relative incidence of these symptoms, collectively known as post-splenectomy syndrome, underscores the relevance of splenic regeneration and functional recovery of the organ as a separate medical problem.

The history of fundamental interest to mammalian spleen regeneration is rather long. From a very early period, studies on the subject split by adherence to one of the two experimental models, involving either resection or heterotopic transplantation of the spleen [5, 6]. The eventual loss of interest in the post-resection in situ splenic regrowth is due to the obvious clinical irrelevance — in practice, virtually all splenic resections are total.

By contrast, regeneration of splenic architecture after heterotopic transplantations is of considerable interest due to the apparent clinical translatability of such procedures [1]. The very idea of spleen autotransplantation has resulted from finding functional fragments of the spleen in the abdominal cavity in some patients after splenectomy. Several explanations for this phenomenon were suggested, from the intrinsic presence of additional spleens (polysplenia) to the spontaneous self-engraftment of splenic fragments during trauma or surgical resection. As the latter cause eventually turned out to be predominant, the phenomenon was termed splenosis [7, 8]. Multiple studies on splenic regeneration in laboratory rodents confirmed the ability of structural recovery for splenic fragments placed in the abdominal cavity and, as turned out later, also under the animal skin [9–11].

The studies featuring heterotopic allogeneic and autologous splenic engraftments capture the dynamics of morphological changes during such regeneration in detail. At the same time, a number of issues regarding these experiments remain unresolved, some of them having definite clinical significance. These issues include first and foremost the dynamics of B and T cell reestablishment in the regenerating splenic grafts, as well as the reconstitution of splenic cell population of monocytes and megakaryocytes.

Besides, regeneration of the spleen after the subcutaneous or intraabdominal autologous transplantations represents a rare example of profound tissue repair in mammals. The unique splenic architecture is recovered *de novo* after the virtually complete demolition. However, cellular sources of this extensive repair remain elusive: it is not clear whether the restoration of the spleen structure occurs by cumulative assimilation of the bone marrow-derived circulating stem cells, which populate the hematopoietic niche newly formed by reticular cells. Another possibility is that the autografts themselves, despite the severity of necrotic changes observed at the early stages of the engraftment, retain a pool of low-differentiated cells with hematopoietic potential.

In this study, we aimed to specify the dynamics of reconstitution of lymphocytes, monocytes and megakaryocytes populations within the subcutaneous splenic autografts, as well as to determine cell sources of spleen regeneration after heterotopic transplantation to subcutaneous locations in mouse model.

## Results

### Restoration of the splenic histological architecture and proliferative activity during the engraftment

**Fig. 1:**
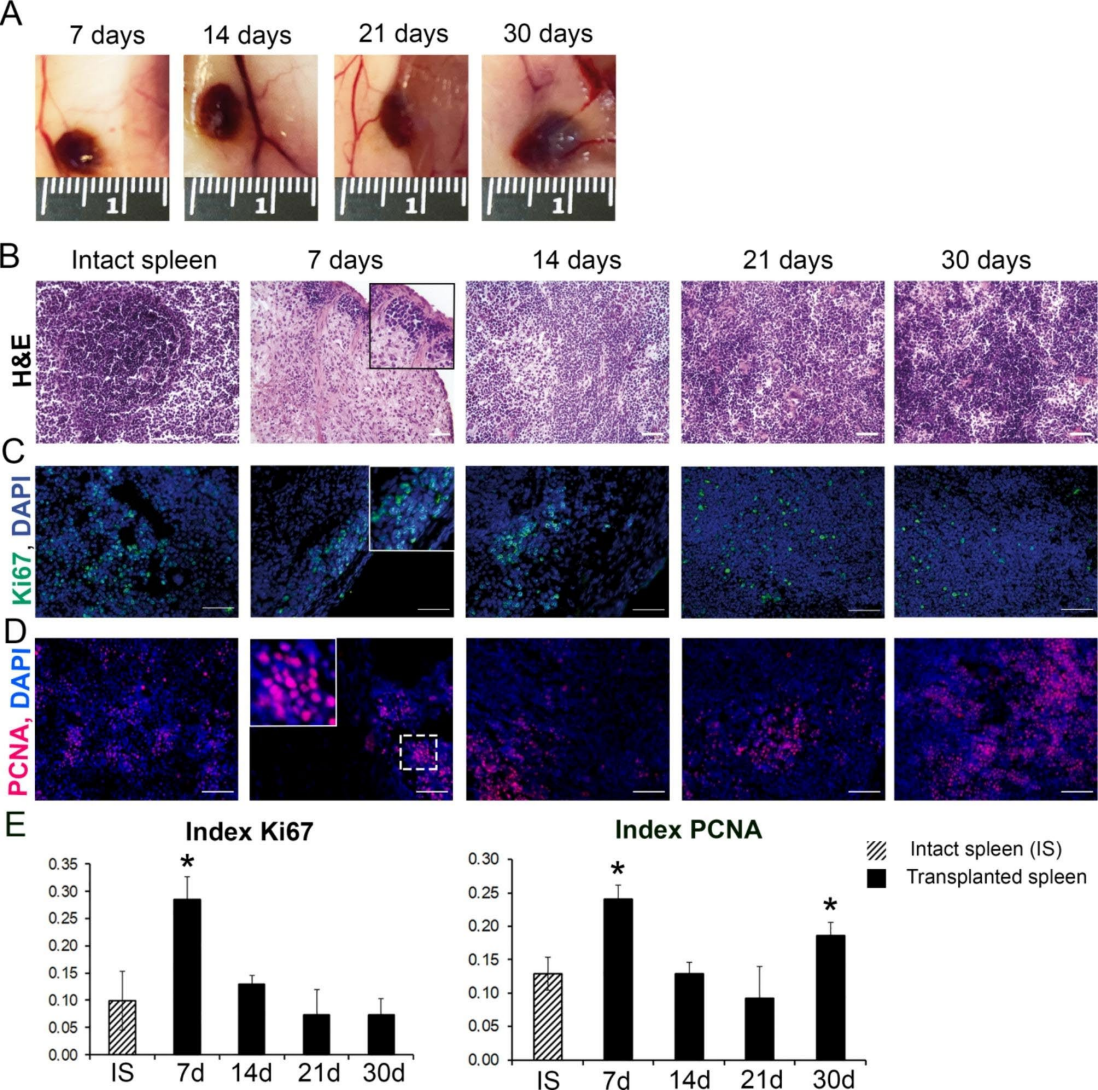
Recovery of the splenic tissue architecture in the transplants. (A) Fragments of the spleen transplanted under the skin. (B) White pulp recovery; hematoxylin and eosin (H&E), objective lens 20, scale bar 100 μm. The proliferation intensity measurements:immunohistochemical visualization using anti-Ki67 antibodies (D) (FITC-conjugated secondary antibodies) and anti-PCNA antibodies (PE-conjugated secondary antibodies) (C), cell nuclei counterstained with DAPI, objective lens 40, scale bar 50 μm. The incision on day 7 was taken from the marginal zone of the graft in which cells with a dark nucleus first appeared. E. Ki67 index and PCNA index dynamics

The transplanted splenic fragments examined on post-transplantation day 5 presented with two clearly identifiable zones: the central zone of necrosis with collapsing lymphocytes and the peripheral zone comprising the de novo forming capsule and stroma. Small accumulations of cells with dark rounded nuclei and thin rims of cytoplasm were observed beneath the newly forming capsule; the number and relative area of such lymphocyte-like cell accumulations increased by post-transplantation day 7. At the same time, in the immediate vicinity of spleen fragments we could always notice the appearance of 1–2 large vessels, from which branches departed to the area of grafts (Fig. 1A,B).

According to immunohistochemical assay, these cell accumulations were almost totally positive for Ki67 proliferation marker (Fig. 1C,E). By post-transplantation day 14, lymphoid cells filled almost the whole volume of the graft and Ki67 + cells formed distinct accumulations among them (Fig. 1C). The segregation between red and white pulp and the onset of lymphoid nodule formation became observable by post-transplantation day 21 and more pronounced by post-transplantation day 30, with Ki67 + cells acquiring a uniform distribution within the newly established splenic architecture (Fig. 1B,C). The dynamics of PCNA expression were similar, with the exception of 30 days, at which the number of PCNA-positive cells was again statistically significantly higher than in the control (Fig. 1D,E).

**Fig. 2:**
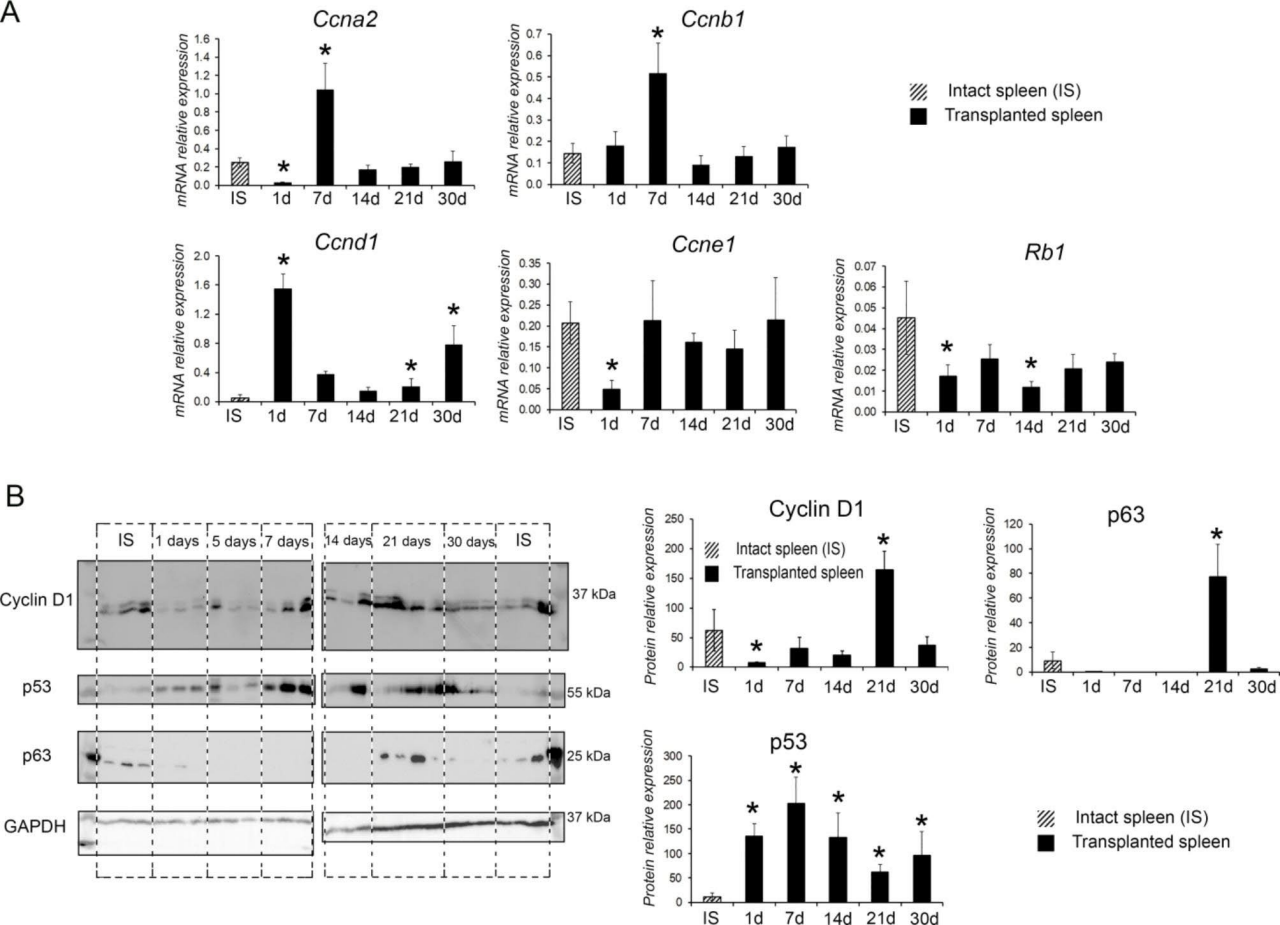
Analysis of gene expression and protein content regulating cell proliferation and cell death in the regenerating spleen. (A) Analysis of expression levels of a cyclins genes in the regenerating splenic transplants. (B) Western blot analysis of proteins regulating proliferation and cell death in regenerating transplants of the spleen. For each term of the study, the experimental group consisted of 5–6 animals. The control group included intact animals (n = 5). The data are presented as means ± standard deviations, * *p* < 0.05 vs. intact spleen (IS).

The activation of cell proliferation processes in the transplanted splenic fragments correlated with the dynamic changes in expression of particular proliferation- and apoptosis-related genes, as well as the content of corresponding proteins.

Transcription levels of cyclins A2 and E1 within the transplants were decreased on post-transplantation day 1, but restored later on and did not differ from the control levels at all subsequent time points, while the activity of cyclin B1 gene significantly increased by post-transplantation day 7 (Fig. 2A). Expression of cyclin D1 gene increased significantly compared with the control as late as by post-transplantation days 21 and 30, which is consistent with the results of western blot analysis showing the maximal content of cyclin D1 within the splenic transplants on post-transplantation day 21 (Fig. 2B).

Expression of Rb1 gene on post-transplantation days 1 and 14 was significantly decreased compared with the control. The p53 protein content was increased at all periods of the study, while p63 was almost absent from the grafts on post-transplantation days 7 and 14 and reached the control levels by post-transplantation day 30. The highest content of p63 protein was found on the 21st day after transplantation (Fig. 2B).

### Reconstitution of the functional cell populations of the spleen within the grafts

**Fig. 3:**
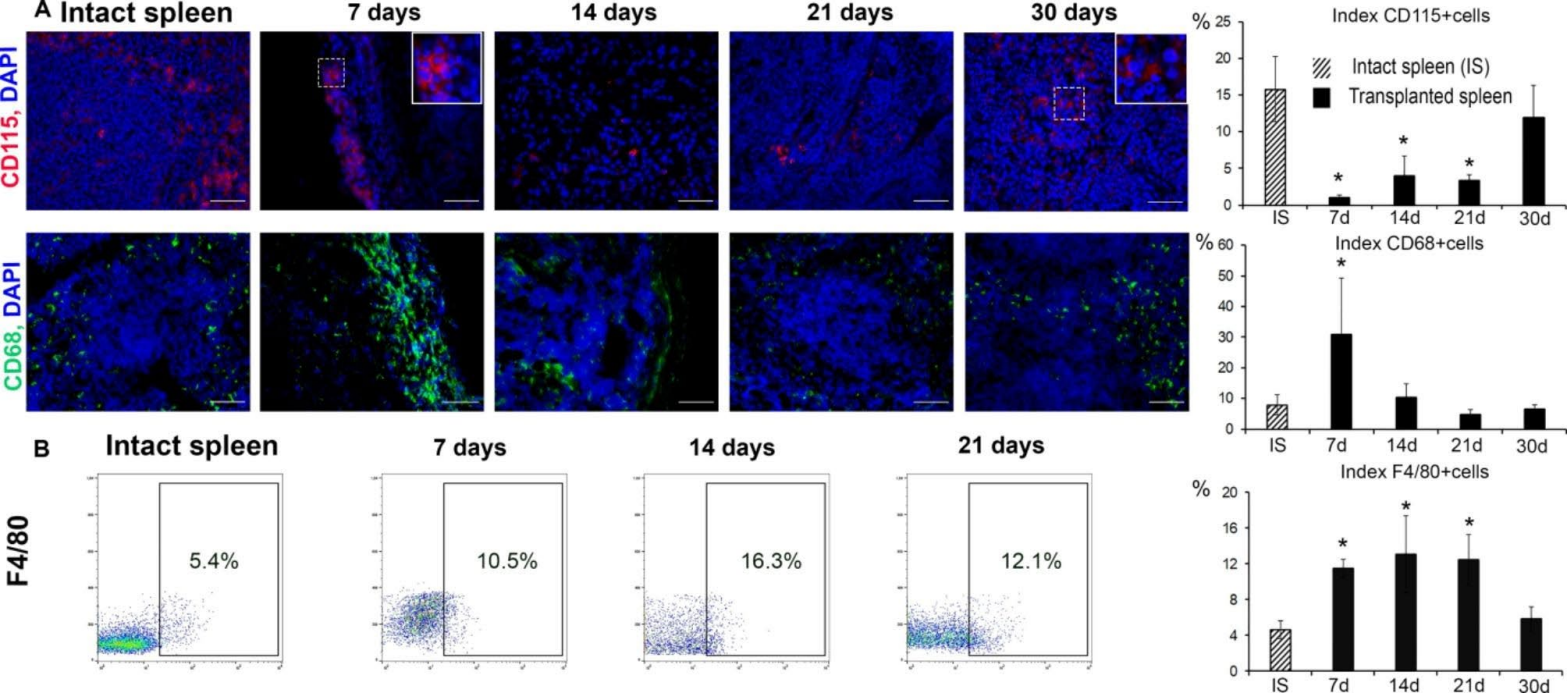
Reconstitution of monocyte-macrophage system in the regenerating splenic grafts. (A) Immunohistochemical visualization of CD115 (PE fluorescence) and CD68 (FITC fluorescence), objective lens 40, scale bar 50 μm. The incision on day 7 was taken from the marginal zone of the graft in which cells with a dark nucleus first appeared; on day 30, the incision demonstrates the spread of CD115 + cells throughout the graft. Reconstitution dynamics for CD115 + and CD68 + cells. The data are presented as means ± standard deviations, * *p* < 0.05 vs. intact spleen. (B) Representative F4/80-SSC scatter plots. Reconstitution dynamics for F4/80 + macrophages. For each term of the study, the experimental group consisted of 5–6 animals. The control group included intact animals (n = 5). The data are presented as means ± standard deviations, * *p* < 0.05 vs. intact spleen

The process of cellular reconstitution within regenerating splenic fragments revealed certain characteristic features. According to the results of immunohistochemical evaluation, the numbers CD68 + macrophages significantly exceeded the control values by post-transplantation day 7 (*p* < 0.05) (Fig. 3A). This probably reflects the process of removing necrotic graft tissues. The number of CD115 + cells increased gradually and reached a maximum on day 30 after transplantation (Fig. 3A). In this regard, we observed a statistically significant decline in CD115 + cell numbers within the grafts on post-transplantation days 7, 14 and 21 (*p* < 0.05) and subsequent replenishment of these cells by day 30 (*p* > 0.05) (Fig. 3A). The flow cytometry assay showed a gradual increase in the number of F4/80 + macrophages as compared with the control, reaching maximum by post-transplantation day 14 (*p* < 0.05) and subsequently returning to the control levels (Fig. 3B).

**Fig. 4:**
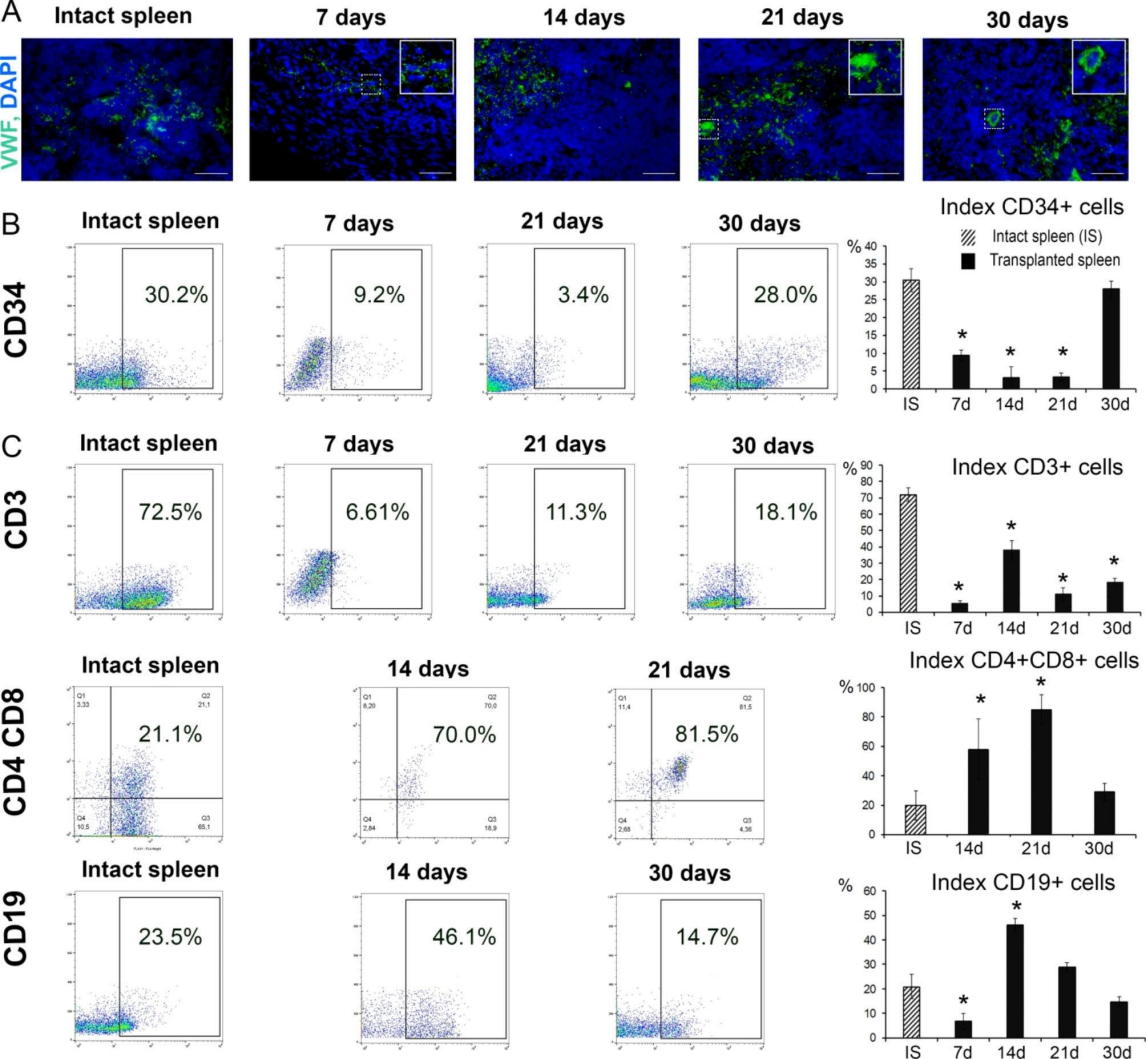
Reconstitution of functional cell types in the regenerating splenic grafts. (A) Immunohistochemical visualization of von Willebrand factor (VWF, FITC fluorescence) positive cells, objective lens 40, scale bar 50 μm. (B) Representative CD34-SSC, CD3-SSC, CD19-SSC scatter plots and CD4CD8 dot plots. С. Reconstitution dynamics for Т and В lymphocytes. For each term of the study, the experimental group consisted of 5–6 animals. The control group included intact animals (n = 5). The data are presented as means ± standard deviations, * *p* < 0.05 vs. intact spleen

We considered megakaryocytopoiesis processes as an indicator for the reconstitution of hematopoietic functionalities by the heterotopically transplanted splenic fragments. Immunohistochemical assessment of von Willebrand’s factor (vW) expression revealed association of this protein with small mononuclear cells on post-transplantation day 7 (Fig. 4A). The occurrence of fine granules of this protein in the vicinity of such cells apparently indicated the formation of platelets already at such early stages of regeneration. By post-transplantation day 14, the grafts presented with contiguous fields of cells and granules stained positively with anti-vW antibodies, as well as solitary vW + giant cells apparently being megakaryocyte precursors. The vW + giant cells considerably increased in number by post-transplantation day 21 and even further by day 30 when their numbers became similar to those observed in intact spleens (Fig. 4A).

Upon reconstitution of lymphocytopoiesis in the heterotopically transplanted splenic fragments, we observed a statistically significant decline in CD34 + cell numbers within the grafts on post-transplantation days 7, 14 and 21 (*p* < 0.05) and subsequent replenishment of these cells by day 30 (*p* > 0.05) (Fig. 4B). The counts of CD3 T cells were significantly lower compared with the control throughout the observation period (*p* < 0.05) (Fig. 4C). At the same time, the smallest number of CD3-lymphocytes was noted on the 7th day after transplantation and amounted to approximately 5% of CD45 + cells. In this regard, at this period, the proportion of subpopulations of CD4 + and CD8 + at these periods was not taken into account in the analysis of the dynamics of these subpopulations of lymphocytes (Fig. 4C). The proportion of CD4 cells was significantly reduced at post-transplantation day 14 and 21 (*p* < 0.05) and converged with the control values later on (*p* > 0.05). By contrast, the proportion of CD8 + lymphocytes cells was significantly increased at post-transplantation day 21 only (*p* < 0.05). The counts of CD4 + CD8 + lymphocytes were also increased significantly at all time points compared with the control (*p* < 0.05). Moreover, it should be noted that CD8 + and CD4 + cells encountered at all time points were mostly double-positive (CD4 + CD8+). The high proportions of double-positive CD4 + CD8 + lymphocytes, about 60%, imply the reciprocally decreased counts of single-positive cells within the grafts (Fig. 4C). The proportion of CD19 + lymphocytes exceeded the control values on post-transplantation days 14 and 21 and subsequently returned to control levels characteristic of the intact spleen (Fig. 4C).

### Expression of genes, previously associated with the regulation of spleen development, within the transplants

**Fig. 5:**
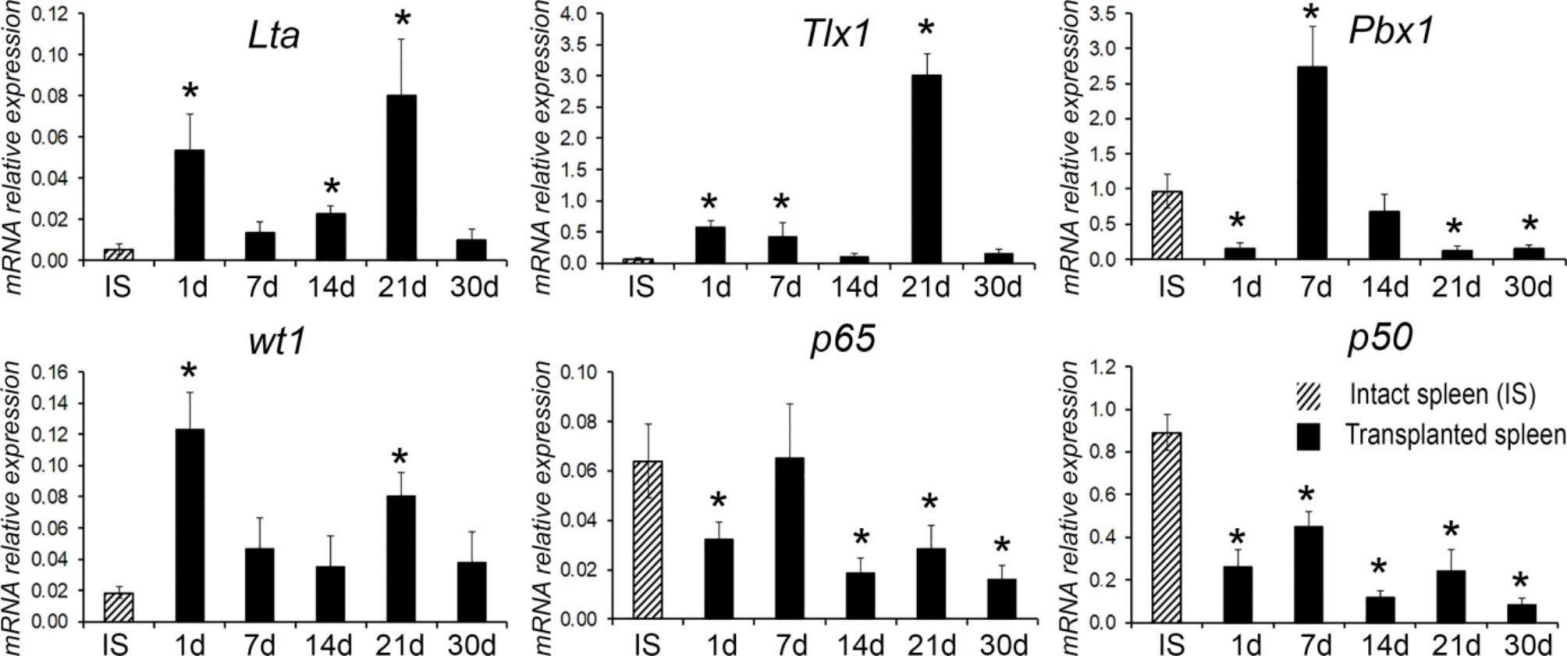
Analysis of expression levels for a set of genes known to regulate splenic growth and development in the regenerating splenic grafts. For each term of the study, the experimental group consisted of 5–6 animals. The control group included intact animals (n = 5). The data are presented as means with the bars for standard deviations.* - statistical significance of differences (as compared with the intact spleen; *p* < 0.05)

Our reconnaissance transcriptomic analysis was focused on key members of signaling pathways involved in prenatal development of the spleen. Transcription levels of lymphotoxin A gene were significantly elevated on post-transplantation days 1, 14 and 21. Significant changes were also observed for several genes known to participate in the early splenic development, including *Tlx1, Pbx1* and *wt1* (Fig. 5). Elevated transcription levels were observed for *Tlx1* on post-transplantation days 1, 7 and 21 and for Pbx1 on post-transplantation day 7, although the activity of *Pbx1* on post-transplantation days 1 and 30 was significantly reduced compared with the control. Expression of wt1 within the grafts was sharply increased on post-transplantation days 1 and 21. At the same time, we observed downregulated transcriptional activity of NFκB signaling pathway genes within the grafts, including reduced expression of *p50* gene on post-transplantation days 1, 14, 21 and 30 and reduced expression of *p65* gene on post-transplantation days 1, 14 and 30 (Fig. 5).

### Cellular sources of heterotopic regeneration of the spleen

**Fig. 6:**
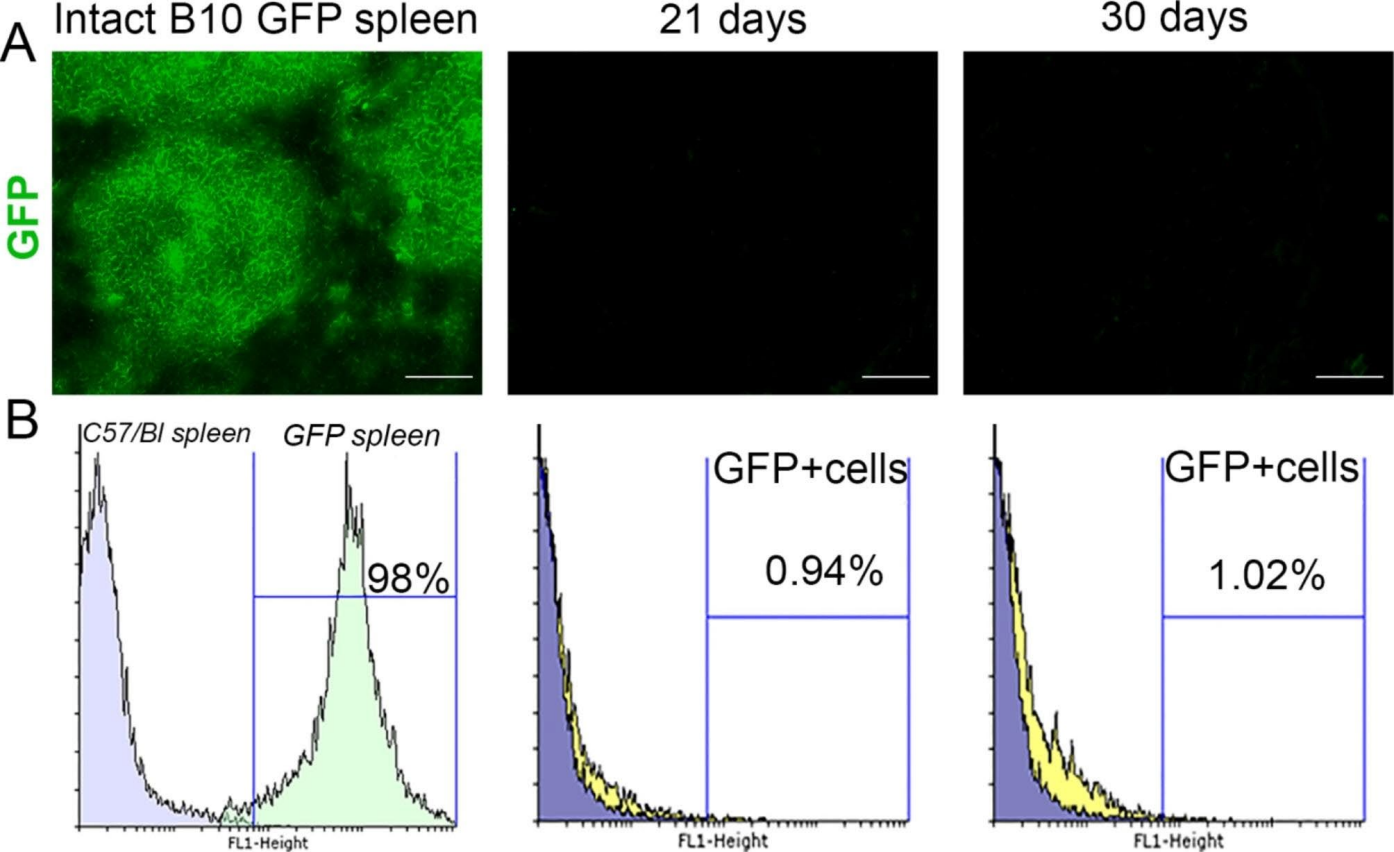
Cellular sources of regenerating splenic parenchyma during the subcutaneous engraftment. (A) Cryosections of the intact spleen of B10-GFP mice and its fragments at different stages of the engraftment, objective lens 40, scale bar 100 μm. (B) Representative histograms of GFP + cells from intact spleen of B10-GFP mice (green histogram), control samples (spleen from C57/Bl mice, blue histogram) and B10-GFP spleen transplants (yellow histogram). For each term of the study, the experimental group consisted of 5–6 animals. The control group included intact animals (n = 5)

To study cellular sources of regeneration of the splenic structures upon heterotopic engraftment we transplanted splenic fragments from B10-GFP male mice to the matching pre-splenectomized C57Bl recipients. On post-transplantation days 21 and 30, about only 1% of the leukocytes within the grafts were GFP+ (Fig. 6A,B).

### Participation of non-cellular and cellular stromal components of the spleen in heterotopic regeneration

Upon decellularization, DNA content of the splenic material decreased to 338 ng/mL, compared with 1674 ng/mL for the intact spleen. The DAPI counterstaining revealed no cell nuclei, which was consistent with the results of hematoxylin and eosin and Mallori stainings applied to this type of material; the sections only presented with collagen fibers of the stromal scaffold of the spleen (Fig. S3).

**Fig. 7:**
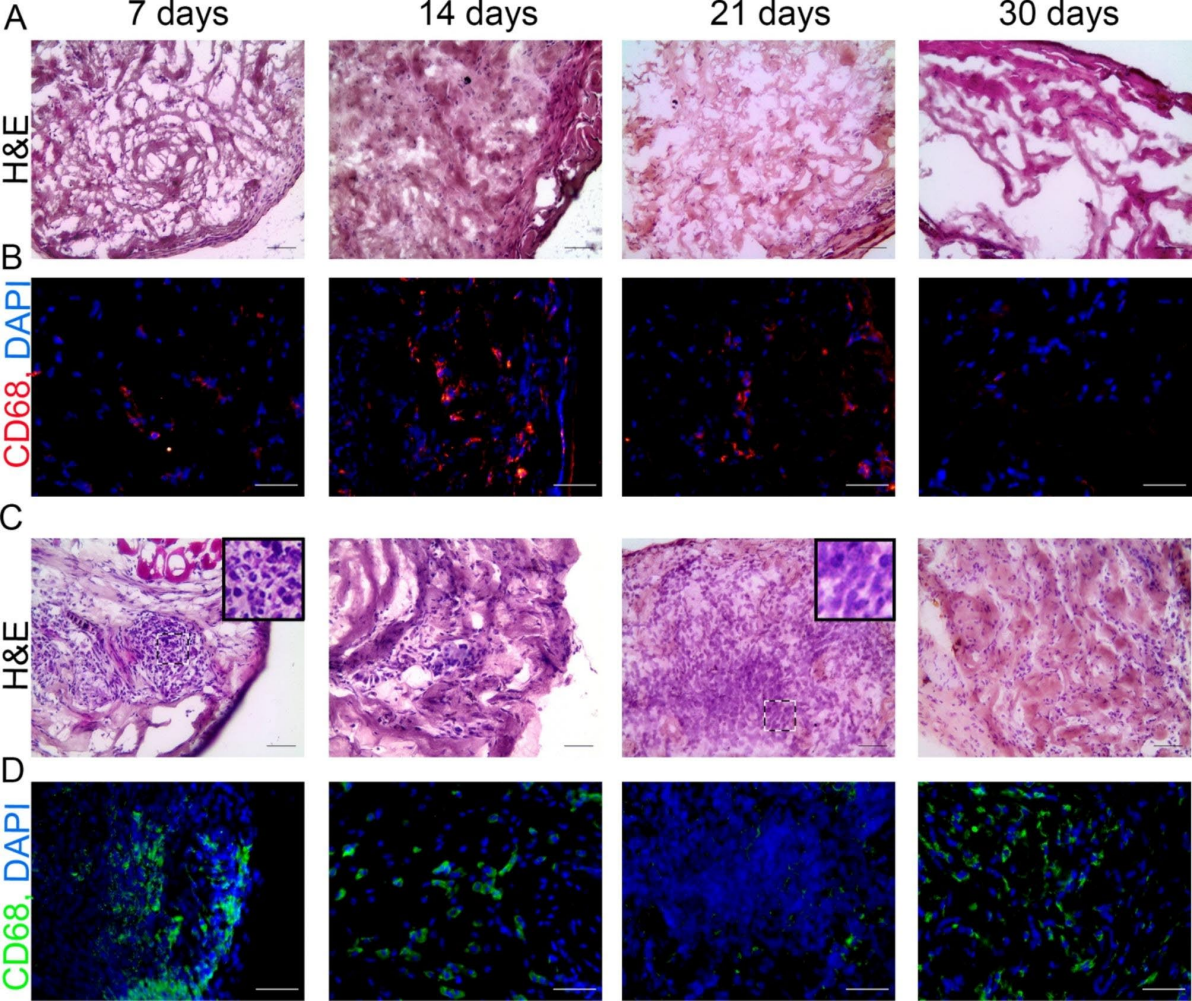
Experiments on the role of stromal component of the spleen in the engraftment. (A) Structural dynamics of a non-repopulated decellularized scaffold upon subcutaneous transplantation; hematoxylin and eosin (H&E), objective lens 20, scale bar 100 μm. (B) Immunohistochemical visualization of CD68 + cells (PE fluorescence) in a non-repopulated decellularized scaffold upon subcutaneous transplantation; cell nuclei counterstained with DAPI, objective lens 20, scale bar 100 μm. (C) Structural dynamics of a decellularized scaffold repopulated with stromal by coculturing upon subcutaneous transplantation; hematoxylin and eosin (H&E), objective lens 20, scale bar 50 μm. (D) Immunohistochemical visualization CD68 + cells (FITC fluorescence) in a decellularized scaffold repopulated with stromal cells by coculturing upon subcutaneous transplantation; cell nuclei counterstained with DAPI, objective lens 20, scale bar 100 μm. For each term of the study, the experimental group consisted of 5–6 animals

The scaffold-transplantation experiments involved three types of scaffolds: (1) decellularized matrix of the spleen, (2) decellularized matrix of the spleen repopulated with splenic stromal cells by injection and (3) decellularized matrix of the spleen repopulated with splenic stromal cells by coculturing. The results of transplantation experiments for the three types of scaffolds were similar: no restoration of splenic architecture was induced by the scaffolds transplanted subcutaneously (Fig. 7A,B,С,D). Throughout the observation period (post-transplantation days 7, 14, 21 and 30), the transplanted acellular scaffolds (non-repopulated) contained fibroblast-like cells and macrophages; the presence of the latter was confirmed by anti-CD68 immunostaining (Fig. 7B,D). The repopulated scaffolds contained clusters of cells with dark nuclei. The majority of these cells were CD68+; at the same time, distinctive white pulp, characteristic of the spleen, was missing (Fig. 7C). Only one of the transplanted repopulated by coculturing scaffolds presented with a large rounded accumulation of lymphocyte-like cells with large nuclei, the majority of which did not express CD68, on post-transplantation day 21 (Fig. 7C,D).

## Discussion

The spleen had long been assigned a secondary role in the human immune system. However, this attitude was questioned by accumulation of clinical data on the long-term consequences of splenectomy. An increasing number of clinical observations indicated a dramatic aggravation in frequency and severity of infectious complications, including sepsis, as well as the overall risks of malignant tumorigenesis, in the aftermath of splenectomy [4]. In connection with these observations, regeneration of the spleen as the means of functional compensation for its loss acquires particular relevance. The lack of *de novo* splenic regrowth after surgical resection is a well-established fact. An alternative, clinically feasible solution came as a result of incidental clinical findings. Some of the patients presented with fragments of fully functional and histologically mature splenic tissue in the abdominal cavity after splenectomy [7, 8]. The discovery of this phenomenon made it a focus of extensive experimental research [9, 10]. Indeed, transplantation of splenic fragments to the greater omentum, as well as to subcutaneous or intramuscular locations, in rabbits, rats and mice facilitated restoration of the characteristic splenic tissue architecture. Nevertheless, despite the structural authenticity of the regenerated structures, their functional competence was not so evident and required additional verification. So far, this issue still has not been resolved. A number of studies have confirmed the ability of heterotopic splenic regenerates to clear the circulation from senescent red blood cells [12, 13] and bacteria, as well as maintain sufficient titers of antibodies in response to immunization with an anti-pneumococcal vaccine [14–16]. In the current study, we confirm restoration of the normal proportion of CD19 + B cells in heterotopic splenic grafts within 30 days, which is consistent with previous findings on the rates of antibody production by transplanted splenic fragments [17]. By contrast with B cells, T cell recovery lasts longer, with the overall proportion of these cells within the graft. Thus, the recovery of T lymphocytes within the graft is not exactly problematic, but definitely more time-consuming. Also, the fact that the regeneration of spleen tissue in transplants continues after 30 days is evidenced by decreased proportion of CD3 + cells on 30 days post-transplantation. This is also consistent with an increased expression of the cyclin D1 gene, a reduced activity of the *p50* and *p65* genes, and an increased content of the p53 protein at this time. These results are consistent with the data obtained in a different model of in situ splenic regrowth after subtotal/partial resections in mice [6].

We were surprised by a large number of double positive CD4 + CD8 + lymphocytes in the regenerating spleen. It is worth noting that in another study with transplantation of spleen fragments irradiated with 5 Gy from 4 day old mice under the kidney capsule of adult 3 month old mice, a large number of CD4 + CD8 + lymphocytes were also observed in the regenerating spleen tissue: after 14 days − 42%, after 3 months − 32% [18]. Hematopoietic stem cells (HSCs) in mice are known to be found not only in the red bone marrow, but also in the spleen. A number of comparative studies of the differentiation potential of such cells have been performed. It was found that during the induction of anaemia in mice, HSCs in the spleen quickly differentiate towards erythrocytes and megakaryocytes [19]. The conditions and severity of differentiation of mouse spleen HSCs into lymphocytes under normal conditions have not been studied, although this possibility is not excluded in principle [20–22]. Probably, such dynamics reflects (high content of CD4 + CD8 + lymphocytes) peculiarities of lymphopoiesis recovery in graft tissue, and also indirectly indicates either differentiation of HSCs of spleen directly into lymphocytes, or migration of the earliest T-lymphocyte precursors into regenerating spleen. It is difficult to say anything about the functional activity and fullness of CD4 + CD8 + lymphocytes of the regenerating spleen. The available data allow us to state that after regeneration finishing the transplant contains functionally complete lymphocytes. This probably concerns both T- and B-lymphocytes. The data indicate that the level of antibacterial immunity and antibody production in response to vaccination in animals with transplanted spleens does not differ from that of control animals (see review [23]). This is consistent with our data on the rapid recovery of the B-population of lymphocytes in transplanted spleen fragments. The aforementioned study on transplantation of irradiated spleen fragments from 3-day-old mice under the kidney capsule of adult mice also noted that after 3 months, lymphocytes obtained from grafts did not differ in their response to activation with LPS or concanavalin A [18].In addition to lymphocytopoiesis, splenic population of megakaryocytes and monocyte-macrophage system are also restored in 30 days post-transplantation, which is important for the regulation of repair processes outside the spleen [24–26]. The sources of recovery of the monocytic-macrophage system in the regenerating spleen remain unclear. There are two possibilities: repopulation by monocytes of bone marrow origin with subsequent differentiation into macrophages, as well as differentiation of the common precursor for monocytes and macrophages found in the spleen in norm [27, 28]. Given that von Willebrand factor is also synthesized in endotheliocytes, an increase in the content of this factor also indicates the growth of the capillary network in the grafts [29].

Apart from its high clinical relevance, splenic grafts that regenerate at heterotopic locations represent a unique model of profound tissue repair in mammals. A unique advantage of this model is that the engraftment involves no specific remodeling of the local blood supply; moreover, the restoration of splenic tissue architecture occurs independently of its heterotopic location within the body. These unique features understandably drive attention to cellular sources of regeneration for this model. In this regard, implications of hematopoietic stem cells, which either persist in the graft or colonize it by gradually arriving from circulation, ascend to early studies [10, 30]. In our experiments with the transplantation of splenic material from B10-GFP mice, in which all cells synthesize the fluorescent tag GFP, to ‘non-fluorescent’ ordinary animals, GFP + cells were almost completely absent from the functional leukocyte fraction of the regenerated splenic material. This important observation implicates the circulating hematopoietic cells, lymphocytes, monocytes and other precursors of the host (or own circulating hematopoietic cells in the case of autotransplantation) as the dominant source of functional splenic reconstitution upon the subcutaneous engraftment. We do not exclude the possibility of graft cell death due to the host immune response. Considering that B10 mice were obtained by interbreeding lines derived from C57BL mice, we believe that an immunological conflict between the donor organism and the host is unlikely. Migration of cells from regenerating spleen fragments from B10 GFP mice to the host organs is not excluded, but we did not investigate this.

Studies using the ablation of hematopoietic cells with ionizing radiation demonstrate the ability of splenic stroma to ensure proliferation and differentiation of the transplanted hematopoietic cells [20, 31, 32]. These studies implicate the native splenic stroma as a key regulatory component promoting regeneration of the hematopoietic parenchyma in this organ. Such a conclusion is consistent with our findings on the elevated expression of genes involved in embryonic development of the spleen and associated with stromal cells — *Tlx1*, *Pbx1*, *wt1* and *Lta* [33–35]. Still, our attempts to induce splenogenesis by transplanting decellularized splenic scaffolds, either pure or repopulated with stromal cells of the spleen, failed. In one case only, 30 days after transplantation of the splenic matrix repopulated with stromal cells, we observed an accumulation of cells resembling lymphoid cells and not expressing CD68. Given the exclusiveness of this observation, it can be attributed to possible contamination of the stromal fraction of the spleen by lymphoid cells during their gradient separation.

The failed attempt to induce the formation of the characteristic splenic tissue architecture may reflect the absence of particular regeneration-related stromal cell phenotypes within the scaffold. It has been demonstrated that prenatal development of lymphoid tissue in lymph nodes and Peyer’s patches requires specifically differentiated non-hematopoietic lymphoid tissue organizer (LTo) cells expressing VCAM-1 + ICAM-1 + MAdCAM-1 + phenotypes [34, 36, 37]. In addition, several studies demonstrate the existence of specific subpopulation of stromal cells with CD31 + MAdCAM-1 + LTbR + phenotype found in the splenic capsule and capable of inducing the formation of splenic parenchyma upon transplantation beneath the renal capsule during the postnatal period [22, 38]. Importantly, this property is retained by these cells within a narrow developmental window at postnatal days 1–3, as long as phenotypically similar cells isolated on postnatal day 8 already lose their capacity to induce splenogenesis [22, 38]. It should be noted that the capacity to regenerate the authentic splenic tissue architecture at heterotopic locations is age-independent, i.e. common for splenic fragments donated by both young and older laboratory animals [23, 39]. Thus, identification of specific populations of splenic stromal cells that induce and organize regeneration of this organ under conditions of heterotopic transplantation remains an open issue.

In addition to the mentioned Tlx1, VCAM1, CXCL12, CSF1, which are also produced by stromal cells [40], are also among the factors involved in the organization in the maintenance of spleen haematopoiesis. It is known that the uptake of aging erythrocytes from the blood stream plays the leading role in the formation of spleen macrophage subpopulations. It was shown that in red pulp macrophages hem of absorbed erythrocytes stimulates the expression of the transcription factor SPIC, which causes an increase in VCAM1 expression. At the same time, HO-1 and ferroportin synthesis are directly stimulated by hem [41]. In addition to the removal of aging erythrocytes, red pulp macrophages are apparently involved in inflammation and immune defense. In response to Plasmodium chabaudi infection, red pulp macrophages secreted large amounts of interferon I [42], and the in vitro ability of this macrophage population to stimulate CD4 + lymphocyte differentiation toward T regulatory cells was also shown [43]. Constant uptake by macrophages of cells dying by apoptosis was found to result in stimulation of expression of scavenger receptors as well as Mertk, Gas6 and CD36 related proteins [44]. Thus, continuous uptake of apoptotic and autoreactive B-lymphocytes maintains the functional activity of germinal centre macrophages, which in turn ensures the correct course of humoral immunity reactions. Metallophilic macrophages of the marginal zone are in close interaction with MadCAM1 + endothelial cells of the marginal sinuses, and the removal of the corresponding subpopulation of macrophages from the spleen leads to a violation of its compartmentalization and disorganization of lymphoid nodules [45], other studies have not found such an effect [46]. It is assumed that B-lymphocytes secrete LTαβ lymphotoxins, which is necessary for the formation of the marginal zone [47]. At the same time, B-lymphocytes themselves migrate to the marginal zone under the influence of oxysterols apparently produced by metallophilic macrophages of the marginal zone [48].

We would like to point out some limitations in our study. We did not study the dynamics of dendritic cell recovery during regeneration of transplanted spleen fragments. Indeed, dendritic cells play a key role in the immune functions of the spleen. During the postnatal period, several populations of CD8α + CD4-CD205+, CD8α-CD4 + CD205-, and CD8α-CD4-CD205- dendritic cells are found in mouse spleens. By the time of birth, the number of dendritic cells in the spleen of mice is extremely low, but increases rapidly and by 5 weeks, after which the composition of the mouse spleen dendritic cell population is virtually unchanged [49]. The role of dendritic cells in the development of typical spleen structure and function is poorly understood. It has been shown that pre-follicular DCs secreting CXCL13 and driving B-cell chemotaxis also contribute to white pulp and marginal zone development after birth along with macrophages [50]. The dynamics of recovery of dendritic cell populations during spleen regeneration has not been studied, but based on the data on the recovery of antibody production by 30 days after transplantation (see review [23]), we can conclude that by this time dendritic cell populations recover as well.

## Conclusions

The results indicate that upon autologous heterotopic transplantation of the spleen to subcutaneous locations, the grafts recover the characteristic splenic architecture within 30 days, which is consistent with other reports. By the end of this period, the graft restores fully functional cell populations representing the monocyte-macrophage system, B lymphocytes and megakaryocytes, whereas the recovery of splenic T lymphocytopoiesis takes longer. The expreriments with cross-strain transplantation of splenic fragments from В10-GFP mice implicate the circulating hematopoietic cells as a principal source of recovery for the splenic lympho- and myelopoiesis. Intrinsic stromal cell lineages within the graft have vital significance for the engraftment, albeit specific phenotypes of the organizer stromal cells among them have yet to be determined.

Regeneration in its manifestation is a very diverse phenomenon and each organism and organ is characterized by its own cytological features. In some cases, the dedifferentiation and proliferation of cells prevails, in others the restructuring of remaining tissues, etc. The peculiarity of the spleen regeneration after fragment transplantation is the presence of the stage of HSCs, lymphocytes and other cells migration, after which the proliferation, differentiation and restoration of the typical histoarchitectonics and spleen function are observed.

## Methods

### Animals

The study involved C57Bl male mice, body weight 20–22 g, obtained from “Stolbovaya” branch of the Federal State Budgetary Institution of Science “Scientific Center for Biomedical Technologies of the Federal Medical and Biological Agency”. The study of cellular sources of the subcutaneous regeneration of splenic fragments involved B10-GFP male mice, body weight 20–22 g, received from the “Andreevka” branch of the Federal State Budgetary Institution of Science “Scientific Center for Biomedical Technologies of the Federal Medical and Biological Agency”. The B10-GFP mouse line is derived from a hybridization of C57BL/10SnY mice and C57BL/6TgN (ACTbEGFP)1Osb mice (Jackson Laboratory).

The animals were housed in plastic cages at 22 ± 1 °C on a 12-hour light/12-hour dark cycle, with the light on from 6:00 am to 6:00 pm and free access to water and standard food for laboratory rodents. The housing conditions complied with the “International recommendations for conducting biomedical research using animals” of 1985, the rules of laboratory practice in the Russian Federation (Order of the Ministry of Health of the Russian Federation of 91/06/2003 No. 267) and the law “On the protection of animals from abuse” chapter V, article 10, 4679-GD of 12/01/1999.

### Animal model

The study included several series of experiments. To study the dynamics of splenic engraftment in mice (n = 53), the spleen was resected under general isoflurane anesthesia. Access to the abdominal cavity was done under sterile conditions just below the xiphoid process of the sternum and the splenic vessels were coagulated. The spleen was dissected into 4 equal fragments. The incision in the muscular layer of the anterior abdominal wall was sutured; pockets were formed beneath the skin to the right and to the left of the incision with a blunt method; fragments of the spleen were positioned in the pockets and the skin was sutured. In general, the course of the operation was similar to that previously described [9].

In order to trace cellular sources of the hematopoietic lineage reconstitution during subcutaneous regeneration of the splenic fragments, splenectomized C57Bl male mice received subcutaneous splenic grafts from the matching B10-GFP donors.

To assess the role of splenic stroma components in the studied model of heterotopic regeneration of the spleen, C57Bl male mice (n = 12) were splenectomized for subsequent autologous transplantation of the decellularized splenic scaffold. The spleens were subject to the decellularization procedure lasting 24 h (see related section). The obtained scaffolds were transplanted subcutaneously to the same donors in the autologous manner. In another series of experiments, decellularized splenic scaffolds were populated with splenic stromal cells for 24 h (see related section) and transplanted subcutaneously to splenectomized donors of the splenic stromal cells in the autologous manner (n = 24).

All interventions were carried out between 10.00 am and 11.00 am. The animals were sacrificed on post-transplantation day 1, 5, 7, 14, 21 or 30 in a CO_2_ chamber. For each term of the study, the experimental group consisted of 5–6 animals. The control group included intact animals (n = 5). The study was approved by the Ethical Review Board at the Avtsyn Research Institute of Human Morphology (Protocol No. 29 (5), November 8, 2021).

### Isolation of leukocytic and stromal cell fractions of the spleen

Isolation of spleen cells was performed according to the protocol described in the literature [51]; the spleen was placed in Hanks’ balanced salt solution (PanEco, Russia) and rinsed with two additional changes of the same solution. The fractions of leukocytes and stromal cells were separated using a 40 μm nylon strainer (SPL LifeScience, South Korea). The stromal tissues were mechanically disaggregated, minced and incubated in a 0.05% solution of collagenases type 1 and 4 (PanEco, Russia) for 25 min at 37 °С on an orbital shaker. The obtained cell suspension was passed through a 100 μm strainer (SPL LifeScience, South Korea); the digestive enzymes were washed off by two changes of Hanks’ balanced salt solution containing 10% of fetal calf serum (PAA Lab., Austria) under centrifugation at 20 °С, 500 g for 10 min. The erythrocytes were lyzed with Red Blood Cell Lysis Solution (Miltenyi Biotec, Germany) in accordance with the manufacturer’s protocol. Cell numbers and viability were assessed using a TC20 analyzer (Bio-Rad, USA). The isolated cells were plated in a fresh culture medium. The purity of the resulting fraction of stromal cells was assessed by flow cytometry; the proportion of CD3 + cells constituted 5–10% (Additional file 1: Fig. S1).

### Decellularization of the spleen

The decellularization was carried out using a protocol developed by Zanardo et al. [52]. The animals were splenectomized under general isoflurane anesthesia as described in Sect. 2.2 and the spleen was placed in Hanks’ balanced salt solution (PanEco, Russia). The organ was perfused with heparin solution (100U heparin/mL PBS) and incubated in 50 mL of the same solution for 3 h at 37 °С, followed by 0.1% SDS at 37 °С for 12 h and 50 mL of bidistilled water at 37 °С for 30 min. The material was subsequently incubated in 50 mL of 1% Triton-Х100 at 37 °С for 1 h, washed in 50 mL of bidistilled water at 37 °С for 30 min and finally in 50 mL PBS at 37 °С for 1 h. The absence of viable cells in the obtained scaffolds was confirmed by DNA concentration measurements and microscopic examination of cryosections stained with DAPI, Mallory stain, or hematoxylin and eosin.

### Repopulation of the decellularized splenic scaffolds with stromal cells of the spleen

The splenic scaffolds were repopulated using two alternative approaches. In the first series of experiments, the stromal cell suspensions (see related section) were injected in the decellularized scaffolds via a 20G needle for subsequent transplantation to pre-splenectomized animals. In the second series of experiments, the stromal cells were cultured with 4 × 4 mm decellularized splenic fragments in bioreactor microtubes containing small volume of DMEM (PanEco, Russia) with 10% fetal calf serum (PAA Lab., Austria) and 1% penicillin-streptomycin (PanEco, Russia) on an orbital shaker (SPL LifeScience, South Korea) placed in a CO_2_ incubator (37 °С, 5% CO_2_). After 24 h incubation, the scaffolds were withdrawn and the repopulation efficiency was assessed by counting the unattached cells left in the bioreactor. The repopulated scaffolds were transplanted to pre-splenectomized animals as described in related section.

### Flow cytometry assay

The isolated cells underwent immunophenotyping for surface markers (CD34, CD45, CD3, CD8, CD4, CD19, F4/80). For immunostaining, 100,000 cells were incubated in 100 µl of Rinsing Solution (Miltenyi Biotec, USA) with primary antibodies at room temperature for 1 h. After incubation, the cells were washed in PBS, resuspended in 0.5 ml PBS, and then transferred to test tubes for analysis in a FACScan flow cytometer (Becton Dickinson, USA) with CellQuest software. In each measurement, 10,000 cells were analyzed. The major cell population excluding debris was demarcated as the region of interest in a dot plot of frontal (FSC) and side scattering (SSC) (Figure A2). The gating strategy for T helpers and cytotoxic T lymphocytes was common and included gating CD45 + cells in the SSC versus CD45 dot plot, then gating a positive population in the SSC versus CD3 dot plot and finally determination of the proportions of CD4 + and CD8 + cells in the CD4 versus CD8 dot plot [53]. B cells were counted in the SSC versus CD19 dot plot after gating of CD45 + cells in the SSC versus CD45 dot plot. The F4/80 + cells were counted in the versus F4/80 dot plot after gating of CD45 + cells in the SSC versus CD45 dot plot. The CD34 + cells were counted after the major cell population gating in the FSC versus SSC dot plot (Additional file 1: Fig. S2).

The assay used CD3e-FITC, CD4-PE-Cy5, CD8a-PE-Cy7, CD19-PE, F4/80-PE, CD45-FITC specific antibodies (eBioscience, USA) and CD34-FITC (Miltenyi Biotec, Germany) in dilutions recommended by the manufacturer.

### Histology and immunohistochemistry assays

Spleen fragments were snap-frozen in liquid nitrogen and cryosectioned; for routine examination, the 5–8 μm thick cryosections were stained with hematoxylin and eosin. For immunohistochemical analysis, the cryosections were incubated for 1 h with the first antibodies, then washed and incubated for 1 h with the fluorophore-conjugated second antibodies (FITC or PE) (1:200, Abcam, UK). Cell proliferation assay used anti-Ki67 antibodies (ab15580, 1:100, Abcam, UK) and anti PCNA antibodies (sc-9857, Santa Cruz, USA). Cell type-specific assays used anti-CD68 antibodies (ab125212, 1:100, Abcam, UK) for generic macrophage populations, anti-CD115 antibodies (Miltenyi Biotec, Germany) for monocytic-macrophageal lineages and anti-von Willebrand’s factor antibodies (ab6994, 1:100, Abcam, UK) for megakaryocytes and platelets; the nuclei were counterstained with 4′,6-diamidino-2-phenylindole (DAPI, Sigma-Aldrich Co LLC). The antibody-stained cryosections were subjected to determination of the positivity index as the ratio of positively stained cells to the total number of observable cells. For each index, at least 3000 cells were included in the observation and the index value was expressed in %.

### PCR assay

Total RNA was extracted from biological material with RNeasy Plus Mini Kit (Qiagen, Germany) and used in cDNA synthesis with MMLV RT kit (Evrogen, Russia). The real-time PCR mixtures were set in duplicates by using qPCRmix-HS SYBR mastermixes (Evrogen, Russia). Gene-specific primers used in the assay are listed in Additional file 1: Table S1. PCR primers for mRNA target. The relative expression levels were calculated using ΔCt approach against *Gapdh* as a reference transcript [54].

### Western blot

To analyze the relative production levels of proteins in spleen samples, we used western blot analysis with chemiluminescent detection. The tissues were homogenized in a protein solubilization buffer from MicroRotofor Lysis Kit (BioRad) supplemented with tributylphosphine (TBP) and a protease inhibitor cocktail. Then the homogenate was centrifugated at 14,000 g for 30 min. The supernatant was collected and mixed with the loading buffer containing β-mercaptoethanol, then heated for 5 min at + 65°C and stored at -20°C. Protein separation was carried out by Laemmli electrophoresis in 10-12.5% polyacrylamide gel [55]. The transfer of proteins to a PVDF (BioRad) membrane was carried out in Trans-Blot Turbo machine (BioRad) at 0.35 A for 45 min. To block the sites of nonspecific binding, the membranes were incubated in EveryBlot Blocking Buffer (BioRad) for 40 min at room temperature. Staining with protein-specific antibodies was performed overnight at + 4°C. After washing, the membranes were placed in a solution of secondary HRP-conjugated antibodies for 1 h at room temperature. The chemiluminescence was visualized with Novex ECL kit (Invitrogen, USA) in ChemiDoc system (Biorad, USA) used according to the manufacturer’s instructions. Analysis of the chemiluminescence intensity distribution was carried out using the ImageLab software with GAPDH as normalization protein. At least 3–6 samples were studied for each time-point after spleen transplantation.

### Statistical analysis

Statistical processing of the data employed the SigmaStat 3.5 software (Systat Software Inc., USA) for one-way ANOVA complemented by post hoc Holm-Sidak test or ANOVA on ranks complemented by either post hoc Tukey test or Dunn’s Method. The differences with *p* < 0.05 were regarded as statistically significant.

## Electronic supplementary material

Below is the link to the electronic supplementary material.

Supplementary Material 1
 Fig. S1. The purity of the isolation of the stromal fraction of spleen cells. Proportion of CD3 + cells. Fig. S2. Gating strategies. Major cell population was placed in the region of interest on dot-plot of frontal (FSC) and side scattering (SSC), excluding debris. The gating strategy for T helpers and cytotoxic T lymphocytes included gating CD45 + cells on SSC versus CD45 dot-plot, then gating a positive population on SSC versus CD3 dot-plot and finally the percent of CD4 + and CD8 + lymphocytes was determined on CD4 versus CD8 dot-plot. B-cells were counted on SSC versus CD19 dot-plot after gating of CD45 + cells on SSC versus CD45 dot-plot. F4/80 + and CD34-positive cells were counted after general gating on FSC- SSC dot-plot. Fig. S3. Decellularized spleen сryosections stained with DAPI (A), Mallory stain, or hematoxylin and eosin (B). Fig. S4. Figure shows the whole blot after cutting membrane at molecular weight 5 kDa-130 kDa, 55 kDa, 35 ~ 40 kDa for Cyclin D1 (37 kDa), P63 (25 kDa), GAPDH (37 kDa), P53 (55 kDa). **Table S1**. PCR primers for mRNA target.

## Data Availability

All data generated or analysed during this study are included in this published article [and its supplementary information files]. Additional data will be made available on request.

## List of Abbreviations

***GFP***: Green Fluorescent Protein
***DAPI***: 4′,6-diamidino-2-phenylindole
***PE***: phycoerythrin
***FITC***: Fluorescein isothiocyanate
***H&E***: hematoxylin and eosin
***TBP***: tributylphosphine
***GAPDH***: glyceraldehyde-3-phosphate dehydrogenase
***IS***: intact spleen
