# Supplementary material for: Spleen regeneration after subcutaneous heterotopic autotransplantation in a mouse model
Source: Biol Res. 2023 Mar 29;56:15. doi: 10.1186/s40659-023-00427-4 (PMC10053607; doi:10.1186/s40659-023-00427-4)
Supplement: Supplementary file 1 — Supplementary Material 1 Fig. S1. The purity of the isolation of the stromal fraction of spleen cells. Proportion of CD3 + cells. Fig. S2. Gating strategies. Major cell population was placed in the region of interest on dot-plot of frontal (FSC) and side scattering (SSC), excluding debris. The gating strategy for T helpers and cytotoxic T lymphocytes included gating CD45 + cells on SSC versus CD45 dot-plot, then gating a positive population on SSC versus CD3 dot-plot and finally the percent of CD4 + and CD8 + lymphocytes was determined on CD4 versus CD8 dot-plot. B-cells were counted on SSC versus CD19 dot-plot after gating of CD45 + cells on SSC versus CD45 dot-plot. F4/80 + and CD34-positive cells were counted after general gating on FSC- SSC dot-plot. Fig. S3. Decellularized spleen сryosections stained with DAPI (A), Mallory stain, or hematoxylin and eosin (B). Fig. S4. Figure shows the whole blot after cutting membrane at molecular weight 5 kDa-130 kDa, 55 kDa, 35 ~ 40 kDa for Cyclin D1 (37 kDa), P63 (25 kDa), GAPDH (37 kDa), P53 (55 kDa). Table S1. PCR primers for mRNA target. [file 40659_2023_427_MOESM1_ESM.docx]

# Spleen regeneration after subcutaneous heterotopic autotransplantation in a mouse model

Andrey Elchaninov^2,3*^, Polina Vishnyakova^1,3^, Anastasiya Lokhonina^1,3^, Viktoria Kiseleva^1^, Egor Menyailo^2^, Maria Antonova^4^, Aiaz Mamedov^4^, Irina Arutyunyan^1^, Galina Bolshakova^2^, Dmitry Goldshtein^5^, Xuhui Bao^6^, Timur Fatkhudinov^2,3^, Gennady Sukhikh^1^

^1^ Laboratory of Regenerative Medicine, National Medical Research Center for Obstetrics, Gynecology and Perinatology Named after Academician V.I. Kulakov of Ministry of Healthcare of Russian Federation, Moscow, Russia

^2^ Laboratory of Growth and Development, Avtsyn Research Institute of Human Morphology of FSBI "Petrovsky National Research Centre of Surgery", Moscow, Russia

^3^ Histology Department, Medical Institute, Peoples' Friendship University of Russia (RUDN University), Moscow, Russia

^4^ Histology Department, Pirogov Russian National Research Medical University, Ministry of Healthcare of the Russian Federation, Moscow, Russia

^5^ Laboratory of Stem Cells Genetics, Research Center of Medical Genetics, Moscow, Russia

^6^ Institute of Therapeutic Cancer Vaccines, Fudan University Pudong Medical Center, Shanghai, China

|  | 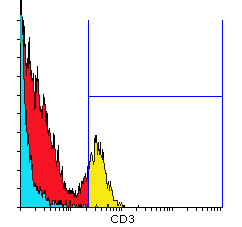  CD3  5.8% |
| --- | --- |

Fig.S1. The purity of the isolation of the stromal fraction of spleen cells. Proportion of CD3+ cells is indicated by yellow colour and below the histogram gate. Red-coloured cells are CD3-. Blue-coloured cells are staining control.


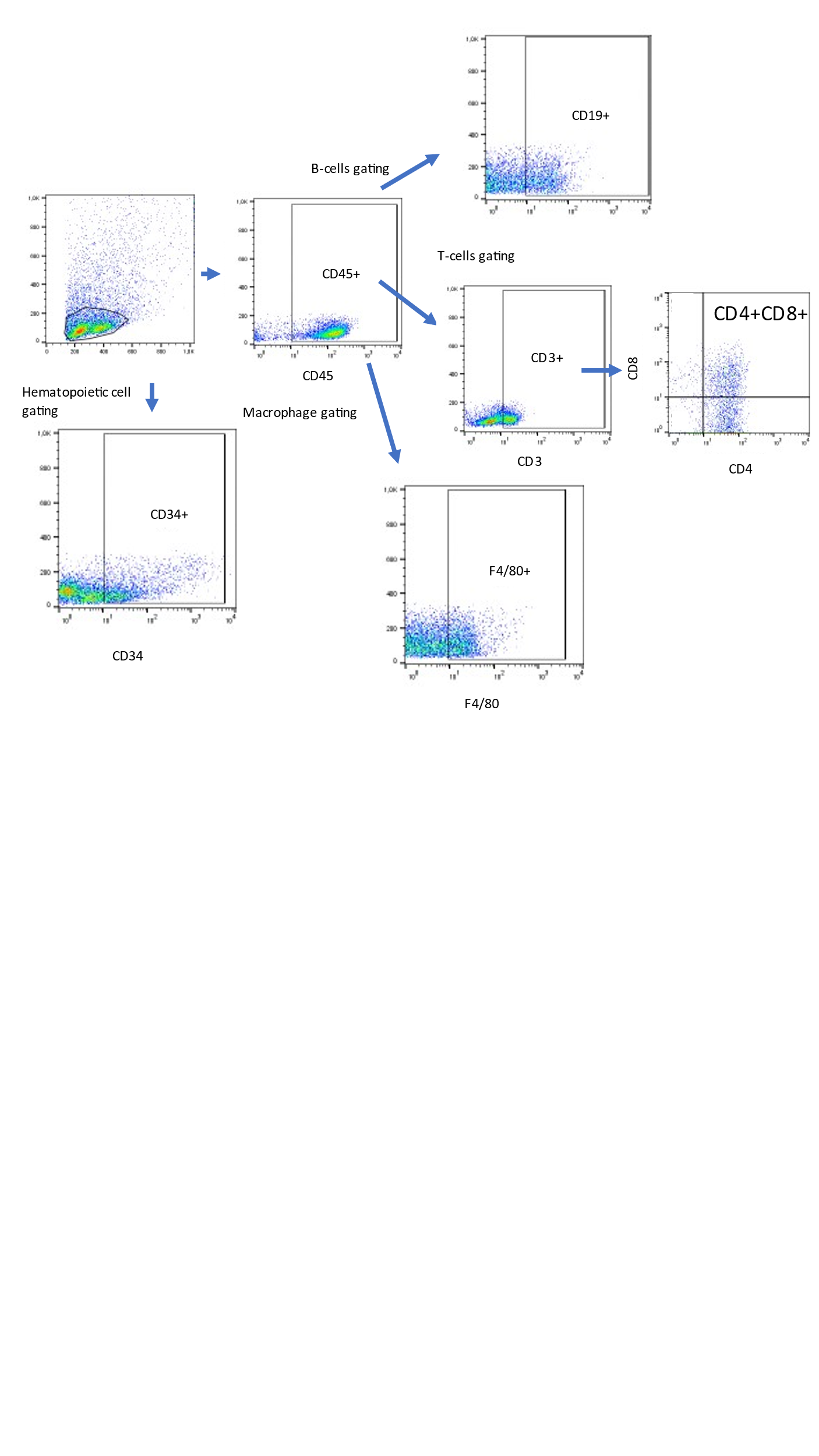


Fig. S2. Gating strategies. Major cell population was placed in the region of interest on dot-plot of frontal (FSC) and side scattering (SSC), excluding debris. The gating strategy for T helpers and cytotoxic T lymphocytes included gating CD45+ cells on SSC versus CD45 dot-plot, then gating a positive population on SSC versus CD3 dot-plot and finally the percent of CD4+ and CD8+ lymphocytes was determined on CD4 versus CD8 dot-plot. B-cells were counted on SSC versus CD19 dot-plot after gating of CD45+ cells on SSC versus CD45 dot-plot. F4/80+ and CD34-positive cells were counted after general gating on FSC- SSC dot-plot.

A

| Intact spleen | Decellularized spleen |
| --- | --- |
|  |  |

B

| Hematoxylin and eosin | Mallory stain |
| --- | --- |
| 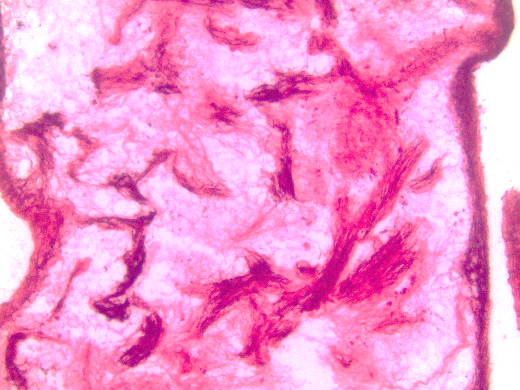 | 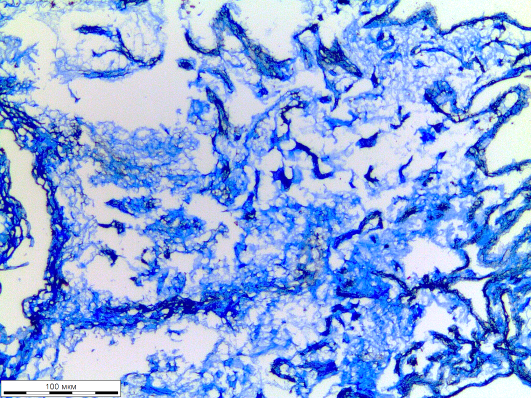 |

Fig. S3. Decellularized spleen сryosections stained with DAPI (A), Mallory stain, or hematoxylin and eosin (B).

**
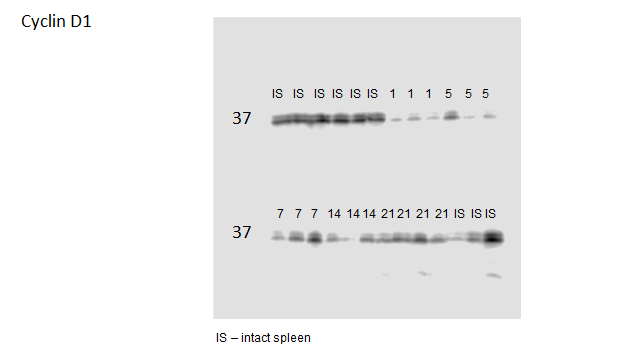
**

**
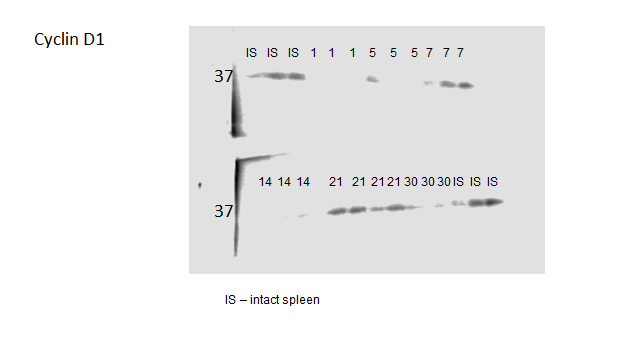
**

**
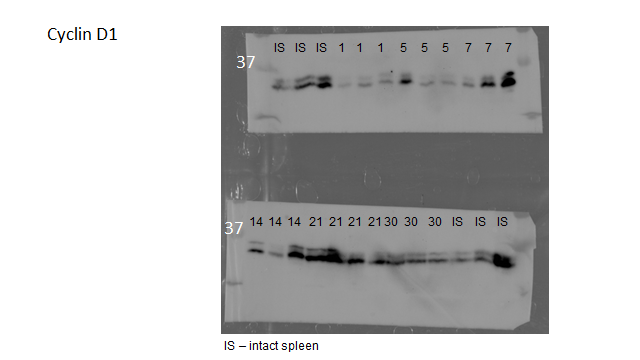

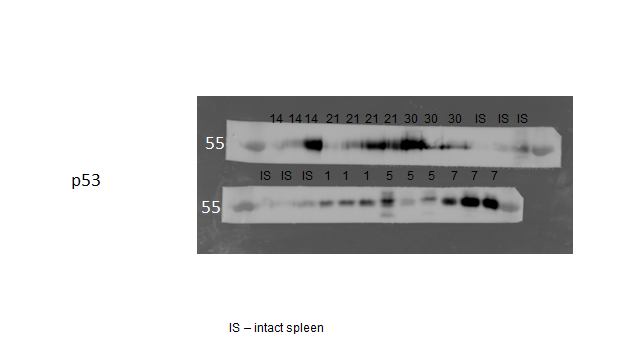

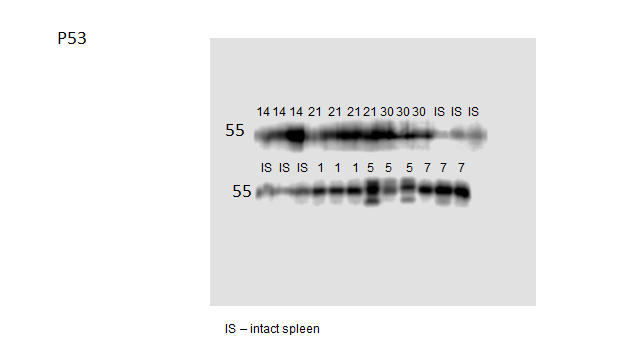

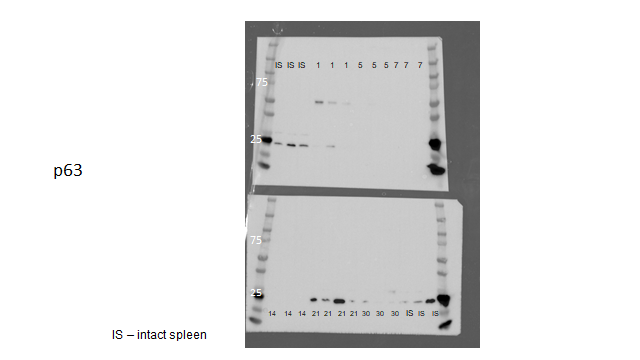
**

**
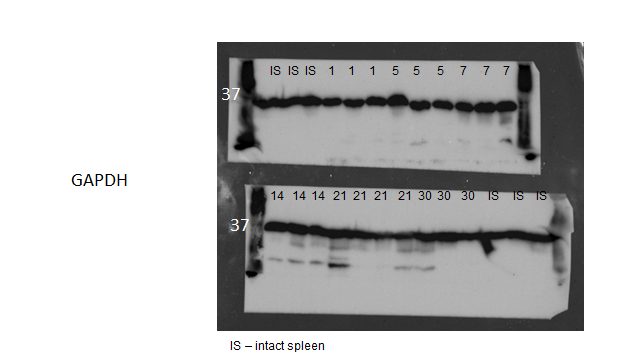
**

**
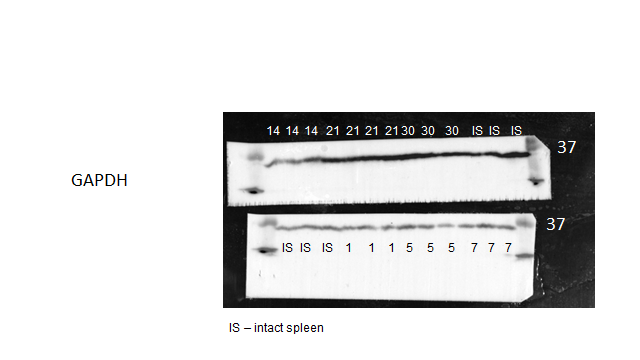
**

**
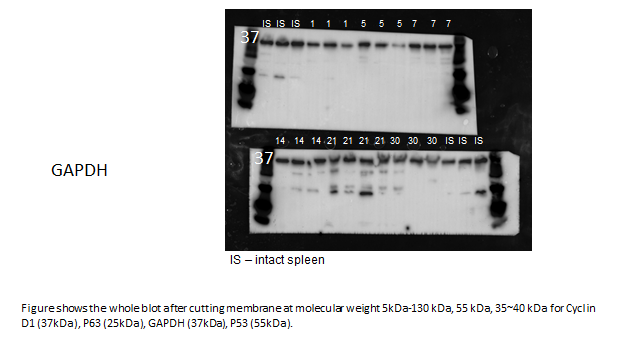
**

Fig. S4. Figure shows the whole blot after cutting membrane at molecular weight 5kDa-130 kDa, 55 kDa, 35~40 kDa for Cyclin D1 (37kDa), P63 (25kDa), GAPDH (37kDa), P53 (55kDa).

**Table S1**. PCR primers for mRNA target

| *Ccna2* | for | TGTCCTGGATTGGGTCACTGG |
| --- | --- | --- |
|  | rev | TCAGCCTCCGGGCAGTAGA |
| *Ccnb1* | for | GCTAAGATCAGCACGCTGGC |
|  | rev | TCGACAACTTCCGTTAGCCTAAACT |
| *Ccnd1* | for | TGTCGGCGCAGTAGCAGA |
|  | rev | AAGATACGGAGGGCGCACAG |
| *Ccne1* | for | TGGATGGTTCCGTTCGCCAT |
|  | rev | GTCAGGACCACACTCGGAGG |
| *Rb1* | for | TGCCTCTCCAGGGTAACCATAC |
|  | rev | GAAGGCTGAGGCTGCTTGTG |
| *Lta* | for | CAGCCCCGACCTAGAACCC |
|  | rev | TGTCATGTGGAGAACCTGCTGTG |
| *p50* | for | AGGTCAAAATTTGCAACTATGTGGG |
|  | rev | CCA GGTTTGCAAAGCCAACCAC |
| *Tlx1* | for | ACAGGTTCACAGGTCACCCC |
|  | rev | TCGGTCATTTTGAGCGCCTTG |
| *Pbx1* | for | GGCATCACAGTCTCCCAGGTATC |
|  | rev | TGTGACAGCCGTTTTGGCAG |
| *Wt1* | for | ACAGAAGGGCAGAGCAACCAC |
|  | rev | AGATACACGCCGCACATCCT |
| *P65* | for | TGCCAGACACAGATGATCGC |
|  | rev | AGTTTCGGGTAGGCACAGCA |
| *Gapdh* | for | AGGCCGGTGCTGAGTATGTC |
|  | rev | TGCCTGCTTCACCACCTTCT |
